# Genome-Wide Analysis of MYB Transcription Factor Gene Superfamily Reveals BjPHL2a Involved in Modulating the Expression of *BjCHI1* in *Brassica juncea*

**DOI:** 10.3390/plants12051011

**Published:** 2023-02-23

**Authors:** Chang Gen Xie, Ping Jin, Jiamin Xu, Shangze Li, Tiantian Shi, Rui Wang, Shuangwei Jia, Zixuan Zhang, Weike Guo, Wenfang Hao, Xiaona Zhou, Jun Liu, Ying Gao

**Affiliations:** 1 State Key Laboratory of Crop Stress Biology for Arid Areas, College of Life Sciences, Northwest A&F University, Xianyang 712100, China; 2 National Key Facility for Crop Gene Resources and Genetic Improvement (NFCRI), Institute of Crop Sciences, Chinese Academy of Agricultural Sciences (CAAS), Beijing 100081, China

**Keywords:** MYB transcription factors, *BjMYB*, *BjPHL2a*, *BjCHI1*, *Brassica juncea*, *Botrytis cinerea*

## Abstract

*Brassica juncea* is an economically important vegetable and oilseed crop. The MYB transcription factor superfamily is one of the largest transcription factor families in plants, and plays crucial roles in regulating the expression of key genes involved in a variety of physiological processes. However, a systematic analysis of the MYB transcription factor genes in *Brassica juncea (BjMYB)* has not been performed. In this study, a total of 502 BjMYB superfamily transcription factor genes were identified, including 23 1R-MYBs, 388 R2R3-MYBs, 16 3R-MYBs, 4 4R-MYBs, 7 atypical MYBs, and 64 MYB-CCs, which is approximately 2.4-fold larger than that of AtMYBs. Phylogenetic relationship analysis revealed that the MYB-CC subfamily consists of 64 *BjMYB-CC* genes. The expression pattern of members of PHL2 subclade homologous genes in *Brassica juncea (BjPHL2)* after *Botrytis cinerea* infection were determined, and *BjPHL2a* was isolated from a yeast one-hybrid screen with the promoter of *BjCHI1* as bait. *BjPHL2a* was found to localize mainly in the nucleus of plant cells. An EMSA assay confirmed that *BjPHL2a* binds to the Wbl-4 element of *BjCHI1*. Transiently expressed *BjPHL2a* activates expression of the GUS reporter system driven by a *BjCHI1* mini-promoter in tobacco (*Nicotiana benthamiana*) leaves. Taken together, our data provide a comprehensive evaluation of *BjMYBs* and show that *BjPHL2a*, one of the members of BjMYB-CCs, functions as a transcription activator by interacting with the Wbl-4 element in the promoter of *BjCHI1* for targeted gene-inducible expression.

## 1. Introduction

*Brassica juncea* is an economically important vegetable and oilseed crop widely cultivated in Asia and Europe [1]. In terms of the well-known “triangle of U” model, *Brassica juncea* is an allotetraploid (AABB, 2n = 4x = 36) improved through interspecific hybridization between the diploid progenitors *Brassica rapa* (AA, 2n = 20) and *Brassica nigra* (BB, 2n = 16), followed by spontaneous chromosome doubling [1,2]. The fungal pathogen *Botrytis cinerea* is a persistent threat to plants throughout the world, including *Brassica juncea*, causing massive yield loss in agriculture [3]. Chitin has been recognized as a general elicitor of plant immune responses, and is also a major structural component of the carbohydrate skeleton of the cell wall of *Botrytis cinerea* [4,5]. Plants have developed a sophisticated system to fight against *Botrytis cinerea* via catalyzing chitin cleavage in the cell wall [6]. Plant chitinases, a class of pathogenesis-related enzymes (PRs), play an essential role in this process via randomly cleaving internal β-1, 4 glycosidic linkages in chitin [6,7].

*BjCHI1* is the first isolated chitinase gene whose expression is associated with defense against *Botrytis cinerea* attack in *Brassica juncea* [8,9,10]. BjCHI1 contains two chitin binding domains (CBDs) [11,12], and extensive investigation has shown that inducible expression of *BjCHI1* is required for defense against *Botrytis cinerea* [11,13]. Several *cis*-acting elements have been predicted in the promoter of *BjCHI1* (BiC-P), and a W box-like 4 element (Wbl-4 box) was recently verified as a fungus-responsive *cis*-acting element [14]. A yeast one-hybrid (Y1H) screen with the core fungus-inducible element as bait was used to identify a number of transcription factors from a cDNA library in *Brassica juncea,* which were isolated and verified [11,15]. BjMYB1, an MYB-CC transcription factor, displayed binding activity to this element and activated the expression of *BjCHI1* [15]. Moreover, Bj26, a C_2_H_2_-type zinc finger protein also displayed binding and activating activity toward the Wbl-4 box [11]. However, the protein factors involved in the hierarchical and dynamic induction of *BjCHI1* remain unclear.

The fungus-responsive Wbl-4 box in the promoter of *BjCHI1* is recognized by BjMYB1, providing novel insight into the transcriptional regulation of *BjCHI1* [11,15]. MYB-like transcription factors are one of the largest transcription factor families in plants, they play important roles in regulating plant growth and protecting plants from various sources of stress, and they are distinguished by the presence of conserved DNA-binding domains named R repeats. MYB transcription factors are thus classified into four subfamilies based on the number of R repeats: R2R3-MYB, 1R-MYB, 3R-MYB, and 4R-MYB [16]. The MYB–CC type transcription factors are a subtype within the MYB transcription factor superfamily that harbor a combination of MYB and coiled-coil (CC) motifs [17]. PHR1 and other MYB-CC genes modulate the phosphate starvation response in plants via binding to the PHR1-binding DNA sequence (P1BS) *cis*-regulatory element within either the 5’ untranslated regions (UTRs) or the promoter regions of phosphate starvation-induced (PSI) genes [17,18,19,20,21,22]. MYB and MYB-CC transcription factor genes have been well characterized in a number of plant species, such as *Arabidopsis thaliana* [16], *Zea mays* [17,23], rice (*Oryza sativa*) [24], *Brassica rapa* [25,26,27], *Brassica napus* [26,27,28], *Hedychium coronarium* [29], *Cymbidium ensifolium* [30], *Apocynum venetum* [31], carrot (*Daucus carota*) [32], mango (*Mangifera indica*) [33], chili pepper [34], and sweet cherry (*Prunus avium* L.) [35]. Nevertheless, relatively few members of the MYB transcription factors in *Brassica juncea* (BjMYBs) have been well explored [15], and there is still no comprehensive study of BjMYBs to date.

Our previous investigation revealed that BjMYB1 plays an important role in mediating the inducible expression of *BjCHI1* by *Botrytis cinerea* and its derived elicitor chitin [15]. In the current study, we conducted a genome-wide exploration of the MYB transcription factor superfamily in *Brassica juncea,* including 23 1R-MYBs, 388 R2R3-MYBs, 16 3R-MYBs, 4 4R-MYBs, 7 atypical MYBs, and 64 MYB-CCs; phylogenetic analysis and the chromosomal location of BjMYBs were determined. In addition, the gene structure and expression profiles of *BjMYB-CCs* were analyzed. Finally, *BjPHL2a* was isolated and verified to be required for inducible expression of *BjCHI1* via interaction with the fungus-responsive Wbl-4 box in the mini-promoter region of *BjCHI1*.

## 2. Results

### 2.1. Genome-Wide Identification of BjMYB Genes

A total of 502 putative BjMYBs with conserved Myb-like DNA binding domains or MYB-CC domains were isolated from the genome of *Brassica juncea*. This is approximatly 2.4-fold more than that of AtMYBs. We found 241 and 230 *BjMYB* genes in the A and B subgenome, respectively (Table S1). The BjMYBs were renamed in terms of their sequence similarity to AtMYBs (Table S1). The protein lengths of BjMYBs were between 96 and 1202 amino acids (aa), with predicted molecular weights (Mw) in the range of 11.58 to 133.39 kDa. The theoretical isoelectric points (pI) of BjMYBs ranged from 4.71 to 10.00 (Table S1). The pI of 309 BjMYBs (60.35%) showed values greater than seven. Other characteristics of BjMYBs, such as gene position and accession number, are presented in Table S1.

### 2.2. Classification and Phylogenetic Analyses of BjMYB Proteins

**Figure 1:**
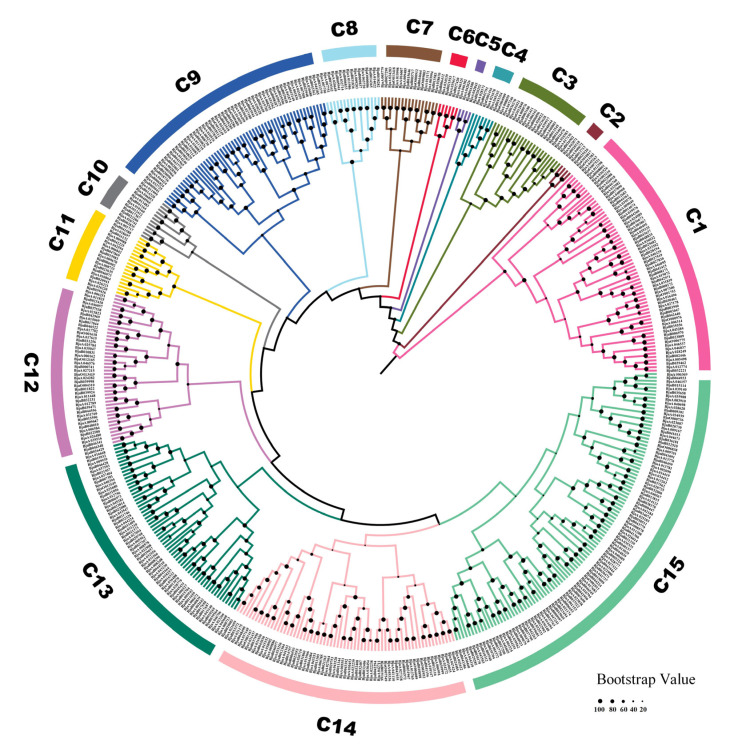
Phylogenetic analysis of MYB transcription factors from *Brassica juncea.* The protein sequences of 502 BjMYB transcription factors were aligned using the MUSCLE tool, and the neighbor-joining (NJ) method (bootstrap replications, n = 1000) was used to generate the phylogenetic tree with MEGA 11. The phylogenetic tree was highlighted with FigTree (version 1.4.4). The proteins are clustered into 15 distinct groups designated by a group number C1~C15 and labeled with different colored branches.

We divided BjMYB proteins into six subfamilies according to the AtMYB nomenclature (Table S1); these divisions include 23 1R-MYBs, 388 R2R3-MYBs, 16 3R-MYBs, 4 4R-MYBs, 7 atypical MYBs, and 64 MYB-CCs. To explore the evolutionary relationships among BjMYBs, an unrooted NJ tree with 502 BjMYBs was constructed. As shown in Figure 1, BjMYB proteins were then classified into 15 groups. Among the groups, groups C9 (56), C13 (61), C1 (64), C14 (66), and C15 (104) were the top largest groups, which represents around 70 % of BjMYB members. Groups C2 and C5 all contained only four members.

### 2.3. Chromosomal Location of BjMYB Genes in Brassica juncea

**Figure 2:**
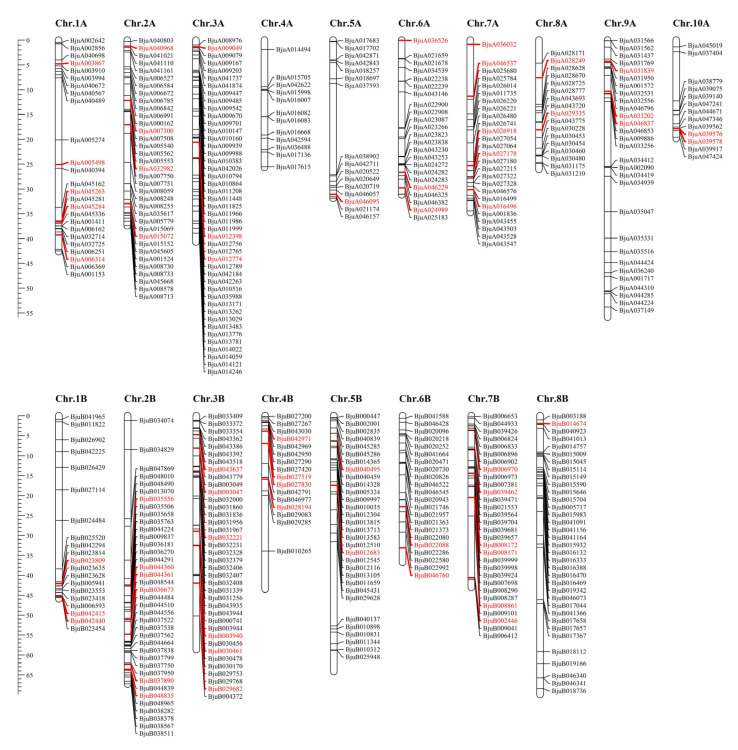
Distribution of the identified MYB transcription factors genes on 18 chromosomes of *Brassica juncea*. Chromosome numbers are indicated at the top of each bar and chromosomal distances are given in Mbp at the left of each bar. The *MYB* genes from the C1 group are labeled in red, while the other members of *MYB* genes are labeled in black.

The chromosomal distribution of *BjMYB* genes was determined with MapChart as previously described [36] and is illustrated in Figure 2. The 471 identified *BjMYB* genes were mapped on 18 chromosomes of *Brassica juncea* (93.82%). Among them, 241 *BjMYB* genes were mapped to the A01 to A10 chromosomes and 230 *BjMYB* genes were mapped to the B01 to B08 chromosomes of *Brassica juncea*. In contrast, there remain 31 *BjMYB* genes on as yet unmapped scaffolds or contigs. *BjMYB* genes were found to be scattered on each chromosome in *Brassica juncea*, but their distribution density differed. Chromosomes A03 (45 members), B02 (38 members), and B03 (38 members) contained a higher density of *BjMYB* genes, while chromosomes A04 (12 members), A05 (16 members), and A10 (13 members) had fewer *BjMYB* genes anchored. We further analyzed the chromosomal distributions of *BjMYB-CC* subfamily genes. As a result, 28 *BjMYB-CC* subfamily genes were found on the A subgenome chromosomes and 30 on the B subgenome chromosomes. Surprisingly, no putative *BjMYB-CC* genes were located on chromosome A04. Moreover, there were six *BjMYB-CC* genes on as yet unmapped scaffolds or contigs.

### 2.4. Gene and Protein Structure Analyses of the BjMYB-CC Genes

**Figure 3:**
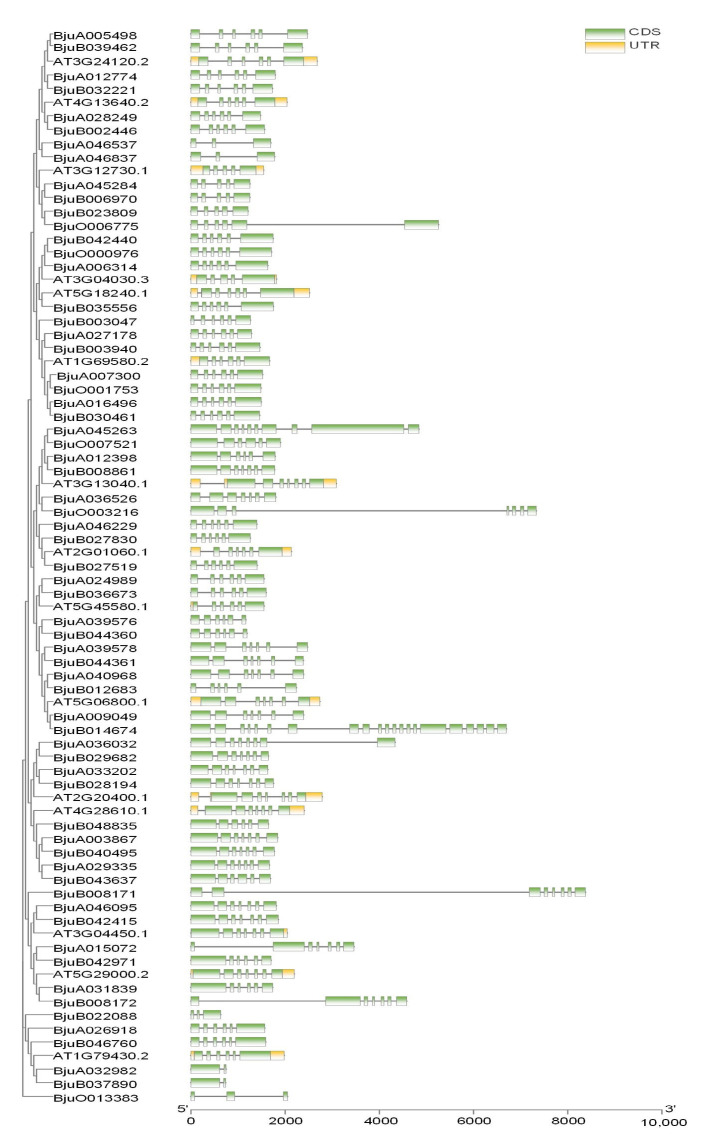
Schematic exon/intron structures of the *MYB-CC* genes from *Brassica juncea* and *Arabidopsis thalina*. Exon/intron organization of MYB-CC genes was visualized using the Gene Structure View (Advanced) from TB tools. The exons and introns are represented by green boxes and black lines, respectively. The UTR regions are indicated with yellow boxes. The length of CDS can be estimated by the scale at the bottom.

We previously identified BjMYB1 that interact with the fungus-responsive *cis*-acting element Wbl-4 box in the promoter of *BjCHI1*, which is an MYB-CC transcription factor [15]. To further explore the phylogenetic relationships within the MYB-CC subfamily, we determined the overall exon/intron profile of *BjMYC-CC* and *AtMYB-CC* genes. Consistent with *AtMYB-CC* genes, all *BjMYB-CC* genes contained at least one intron. The exon and intron organization of *BjMYB-CC* genes was complicated (Figure 3). BjMYB48 (BjuB014674) contained the most introns (20 introns), while BjMYB23 (BjuA032982) and BjMYB20 (BjuB037890) had the fewest introns (one intron each). All of the four members of *BjPHL2* clade (*BjPHL2a*, *BjPHL2a1*, *BjPHL2b*, and *BjPHL2b1*) showed similar exon/intron arrangements to AtPHL2 (Figure 3), suggesting they may have resulted from gene duplication.

**Figure 4:**
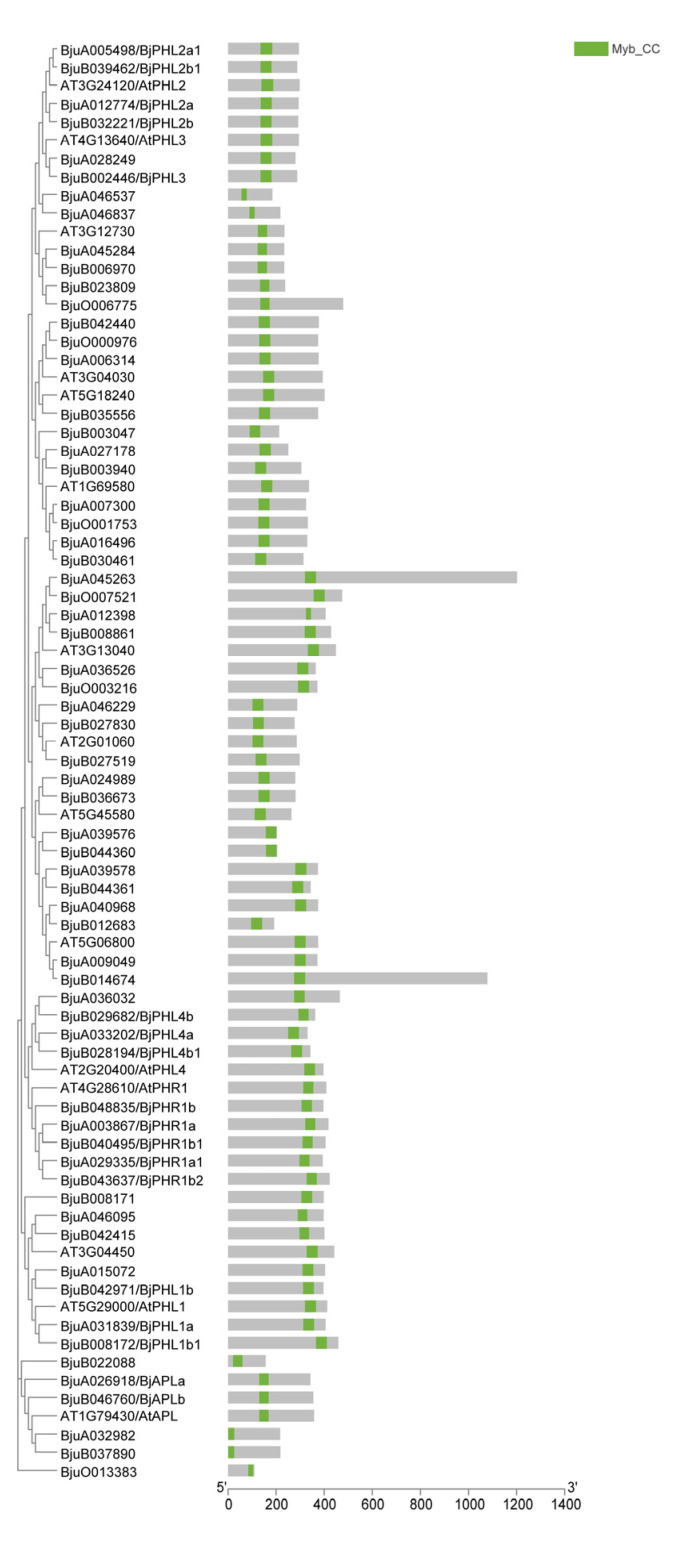
Protein architecture of the MYB-CC proteins in *Brassica juncea* and *Arabidopsis thalina*. The distribution of the conserved MYB-CC motif is represented by green-colored box. See Table S3 for detailed motif information.

We next constructed phylogenetic trees of AtMYB-CCs and BjMYB-CCs (Figure 4), and found that all 64 putative BjMYB-CC transcription factors were named according to their sequence similarity to the corresponding AtMYB-CC orthologues (Figure 1 and Table S1). There were four proteins (BjuB039462, BjuA005498, BjuA012774/BjPHL2a, and BjuB032221/BjuPHL2b) categorized into the same branch as AtPHL2 in our phylogenetic tree; BjuB039462 and BjuA005498 were designated as BjPHL2b1 and BjPHL2a1, respectively. We also observed that BjMYB1 was categorized into the same clade as BjPHL2b1 (Figure 4 and Figure S1). Moreover, the coding sequence (CDS) of BjMYB1 displayed high sequence identity (~98%) to BjPHL2b1 (Figure S2). Compared with BjPHL2b1, BjMYB1 was found to be missing 213-nt in the 5′ region, a 9-nt insertion in the central region, and a G to A replacement at the 3′ end, which resulted in a 71-amino acid-long deletion at the N-terminal and a YGQ insertion in the central region (Figures S1 and S3). To further investigate the evolutionary relationships, we found a total of 10 motifs (with E-value cutoff <e-1.0) [36], including the conserved MYB domain (motif seven), in BjMYBs (Figure S4 and Table S2). The distribution of the conserved MYB-CC motif in each member of MYB-CCs (15 AtMYB-CCs and 64 BjMYB-CCs) was also depicted individually (Figure 4 and Table S3). Consistent with the gene architecture, nearly all of the homologous proteins in the phylogenetic tree of the BjMYB-CC proteins displayed similar motif arrangement to their AtMYB-CC counterparts, such as the four members of *BjPHL2* clade (Figure 4 and Figure S4).

### 2.5. Expression Profiles of BjMYB-CC Genes in Response to Abiotic Stresses

**Figure 5:**
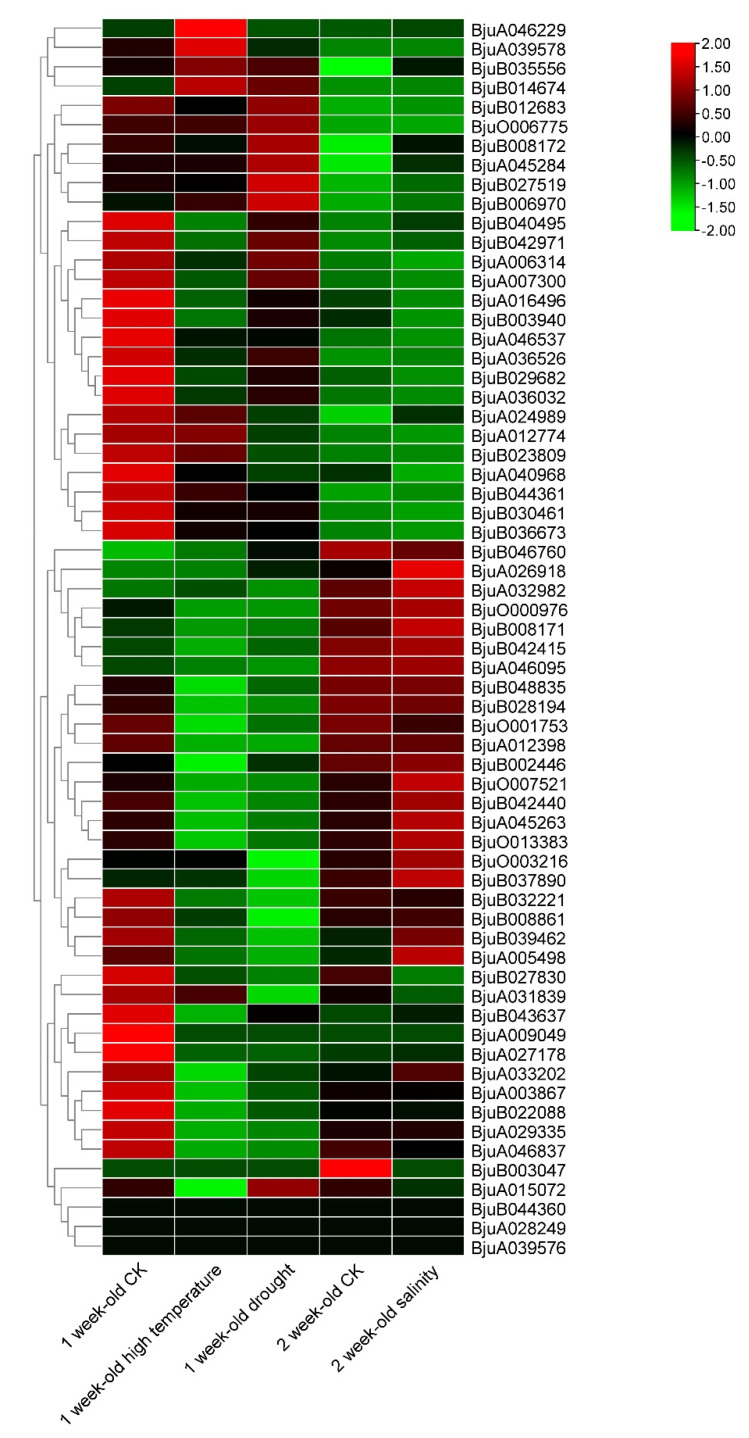
Expression profiles of *BjMYB-CC* genes under different stress conditions. Transcript abundance was normalized according to TPM (transcripts per million mapped reads) values, wherein the TPM values were log2 transformed. The color scale represents relative expression levels from low (green-colored) to high (red-colored).

We next investigated the transcript abundance of 64 BjMYB-CC genes in response to abiotic stresses. The expression profiles under drought, high temperature, and salt conditions were determined using publicly available *Brassica juncea* RNA-seq data [37]. Surprisingly, only a few *BjMYB-CC* genes were observed to be induced in response to specific treatment (Figure 5 and Table S4). However, many genes were observed to be repressed in response to drought or high temperature treatment. The expression of *BjMYB-CC* genes under salt treatment displayed results similar to those of the control treatment (Figure 5 and Table S4).

### 2.6. Expression of BjPHL2 Subclade Genes after Botrytis cinerea Infection

**Figure 6:**
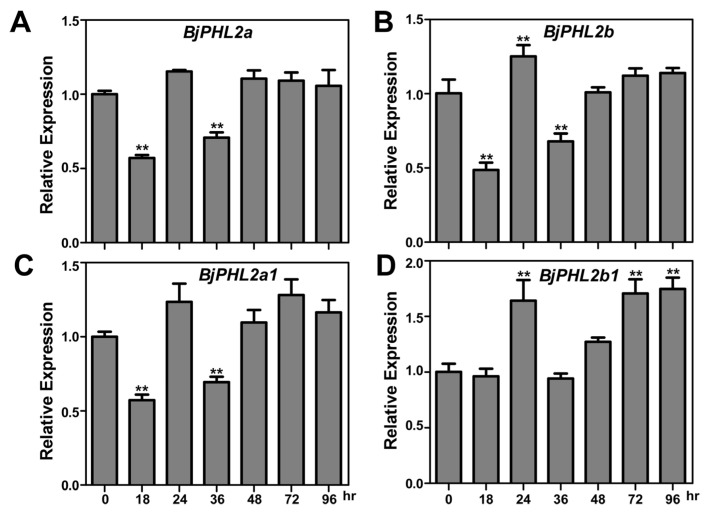
Expression of *BjPHL2* subclade genes in response to *Botrytis cinerea* infection. (**A**–**D**) qRT-PCR analysis of the expression pattern of *BjPHL2a, BjPHL2b, BjPHL2a1,* and *BjPHL2b1* in response to *Botrytis cinerea* infection. Seedlings of *Brassica juncea* were infected with *Botrytis cinerea* followed by sampling at 0, 18, 24, 36, 48, 72, and 96 hours after infection. The relative expression levels of *BjPHL2a, BjPHL2b, BjPHL2a1,* and *BjPHL2b1* were normalized to the expression of BjuB025449 (an AtACT2-like gene in *Brassica juncea*).

As mentioned above, there were four BjMYB proteins categorized into the same branch as the previously identified BjMYB1 in our phylogenetic tree (Figure 4). BjMYB1 was reported to be involved in host defense against fungal attack through activating the expression of *BjCHI1* by binding to the Wbl-4 element in the promoter [15]. It is tempting to speculate that gene expression patterns can provide important clues for paralogous genes function [33,36]. In order to give insight into the role of *BjPHL2* subclade members in plant resistance against fungal infection, we examined the expression of *BjPHL2a* and its paralogs after infection by *Botrytis cinerea*. Quantitative real-time PCR (qRT-PCR) analysis showed that only the transcription of *BjPHL2b1* was slightly induced by *Botrytis cinerea*, reaching a peak at 24 hours after inoculation (Figure 6). The expression pattern of *BjPHL2a1* and *BjPHL2b* after infection by *Botrytis cinerea* were similar to that of *BjPHL2a* (Figure 6), all of which were not significantly induced by *Botrytis cinerea* (no more than 1.5-fold).

### 2.7. Isolation of BjPHL2a

**Figure 7:**
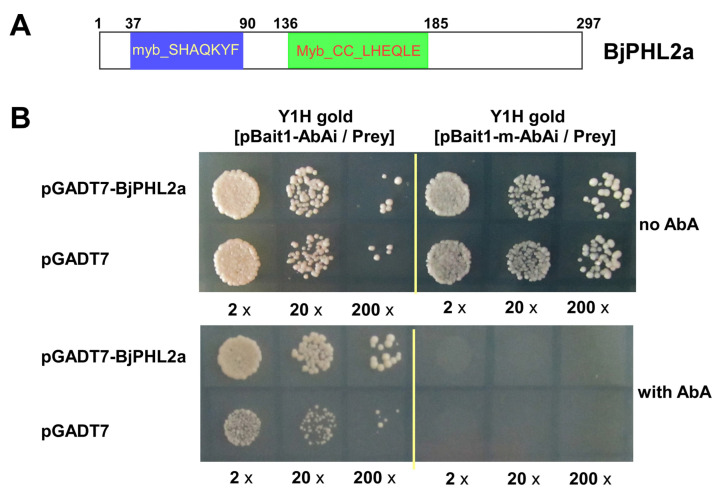
*BjPHL2a* interacts with the Wbl-4 element from *BjCHI1* promoter. (**A**) Schematic diagrams of *BjPHL2a*. The deduced 297 amino acids and the putative two MYB domains (in bold) of *BjPHL2a*. The myb_DNA binding motif (myb_SHAQKYF) is shown in the blue box; the MYB-CC domain (Myb_CC_LHEQLE) is shown in the green box. The numbers above the box indicate the amino acid positions in *BjPHL2a*. (**B**) Yeast one-hybrid analysis of *BjPHL2a*. The bait-reporter yeast strain Y1Hgold [pBait1-AbAi] (Bait) and bait mutated strains Y1Hgold [pBait1-m-AbAi] (Bait-m) expressing the indicated plasmids were grown on SD/-Leu (no AbA) and SD/-Leu/+AbA^550^ (with AbA) agar plates, respectively. Yeast cells were incubated until the optical density at 600 nm reached 0.8 and then were diluted 2-fold (2×), 20-fold (20×), or 200-fold (200×) and used for assays.

We previously performed systemic Y1H screening of a cDNA library from *Brassica juncea* to identify putative transcriptional factors that interact with the fungus-responsive *cis*-acting element Wbl-4 box in the promoter of *BjCHI1* [11,14,15]. Sequencing analysis revealed that two of the isolated positive clones harbored BjcDNA (BjuA012774), which encodes a predicted protein with 297 amino acids (Figure 7A; 32.5 kDa, pI of 6.55). The CDS of BjuA012774 was used as a query for a local BLAST (basic local alignment search tool) search in *Brassica juncea* CDSs downloaded from the BGD (Brassica Genomics Database) [38]. BjuA012774 displayed high sequence identity (92%) to BjuB032221 [38]. The protein sequences of BjuA012774 and BjuB032221 were used as a query for a BLAST search in TAIR (The Arabidopsis Information Resource). BjuA012774 and BjuB032221 showed high sequence similarity to AtPHL2 (PHR1-LIKE2, At3g24120), which is an MYB-CC type transcription factor involved in the phosphate starvation response [18,39]. The online tool CD-Search was used to identify conserved domains in *BjPHL2a*. As shown in Figure 7A, *BjPHL2a* contains an Myb-CC motif followed by the conserved Myb DNA-binding motif (myb_SHAQKYF). To validate whether *BjPHL2a* actually possesses DNA binding and transactivation activity with the Wbl-4 box mini-promoter, we used a Y1H assay. The full-length coding region of *BjPHL2a* was introduced into the vector pGADT7, which contained a GAL4 activation domain. The resulting pGADT7-BjPHL2a plasmid was then transformed into the bait-reporter yeast strain Y1Hgold [pBait-AbAi] and mutated bait-reporter yeast strain Y1Hgold [pBait-m-AbAi], and growth of the transformants was observed. All of the transformants grew well on SD/-Leu (no AbA) medium (Figure 7B, upper panel). However, only yeast cells containing pGADT7-BjPHL2a grew well on the selective medium—SD/-Leu/+AbA^550^ (SD/-Leu media containing 550 ng/mL AbA) agar plates (Figure 7B, lower panel). Almost no DNA binding and transactivation activity were observed in transformants where the bait-reporter yeast strains [pBait-m-AbAi] were transformed with *BjPHL2a* (Figure 7B, lower panel). Taken together, these results suggest that *BjPHL2a* binds to the Wbl-4 box.

### 2.8. BjPHL2a Encodes an MYB-CC Protein Located in the Nucleus

**Figure 8:**
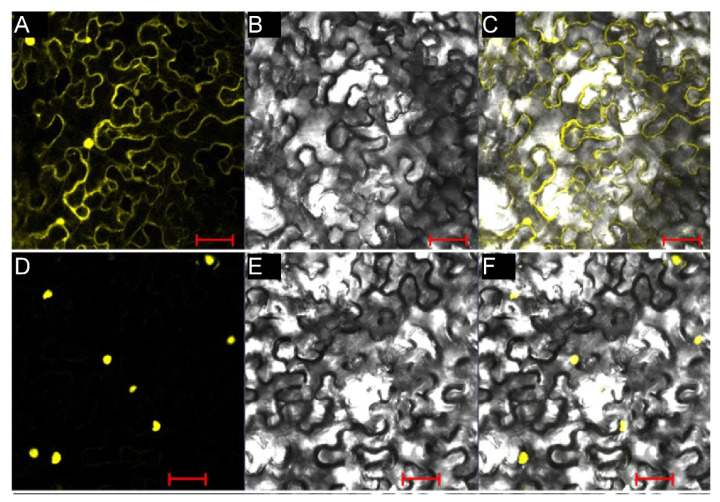
Subcellular localization of *BjPHL2a*. (**A**–**C**) YFP fluorescence from pCAMBIA1205-YFP empty vector. (**D**–**F**) YFP fluorescence from pCAMBIA1205-YFP-*BjPHL2a*. (**A**,**D**) YFP fluorescence in a dark field. (**B**,**E**) Cell morphology in a bright field. (**C**,**F**) Overlay of bright-field and YFP signals. Scale bars = 50 µm.

Sequence analysis revealed that *BjPHL2a* has the same motif arrangement as AtPHL2, including an MYB DNA binding motif at the N-terminus and an MYB-CC motif in the central region (Figure 7A). However, no predicted NLS was observed in *BjPHL2a*, which is a typical characteristic of many transcription factors. To understand whether *BjPHL2a* is a functional transcription factor, we set out to determine the subcellular localization of *BjPHL2a*. The chimeric expression vector pCAMBIA1205-YFP-BjPHL2a and the control vector pCAMBIA1205-YFP were transiently expressed in *Nicotiana benthamiana* leaves via agroinfiltration. We observed fluorescent signals of YFP-BjPHL2a in epidermal cells by confocal fluorescence microscopy. As shown in Figure 8, the YFP-BjPHL2a fusion protein was found to localize predominantly in the nucleus of the cell, while YFP alone was present throughout the cell. These data indicate that *BjPHL2a* is a nuclear-localized protein, consistent with most transcription factors being present in the nucleus.

### 2.9. BjPHL2a Binds to the Wbl-4 Element In Vitro

**Figure 9:**
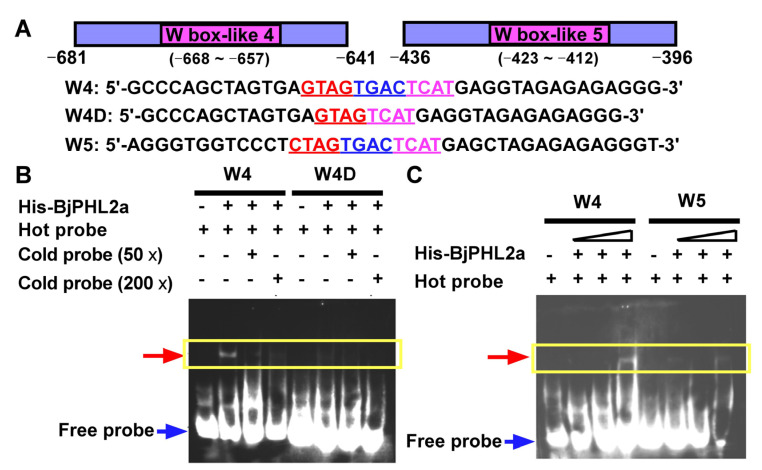
*BjPHL2a* binds to the Wbl-4 element in EMSA. (**A**) Diagrams of probes W4, W4D (W4 mutant), W5, and their nucleotide sequences. W4 contains Wbl-4 element. W4D is a W4 mutant lacking four core base of Wbl-4 element. W5 contains Wbl-5 element. Numbers indicate the nucleotide positions in *BjCHI1* promoter. (**B**,**C**) Electrophoretic mobility shift assays (EMSA) were performed with biotin-labeled (hot probe) or unlabeled probes (cold probe) and recombinant His-BjPHL2a proteins. Specific combinations are shown indicated by a yellow box. An equal amount of *BjPHL2a* protein or hot probe was used in all lanes. Cold probes were added in 50-fold (50×) or 200-fold (200×) excess as binding competitors. The bound and free hot probes are indicated by arrows on the left.

Previous work has demonstrated that the MYB-core element [C/T]NGTT[G/A], AC-elements (ACC[A/T]A[A/C][T/C] and ACC[A/T][A/C/T][A/C/T]), and *cis*-regulatory elements are recognized by R2R3-type MYB transcription factors [15,16]. The MYB-CC protein BjMYB1 binds to W-box or W-box-like elements, but not to AC elements. To determine whether nuclear-localized *BjPHL2a* actually possesses DNA binding activity, we used an electrophoretic mobility shift assay (EMSA). His-tagged *BjPHL2a* recombinant protein was purified and used for EMSA. A shifted band was observed when His-*BjPHL2a* was incubated with a labeled Wbl-4 box (hot probe, Figure 9A, W4). This shift could be competed out with a huge amount (50× or 200×) of non-labeled Wbl-4 element (cold probe, Figure 9A, W4), indicating that *BjPHL2a* is capable of binding to the Wbl-4 element. Furthermore, when the Wbl-5 box was used, almost no shifted band was observed (Figure 9B, W5). In contrast, when the TGAC core sequence of Wbl-4 element was removed, the shifted band was not observed, indicating that deletion of the core sequence TGAC abolished the binding of *BjPHL2a* to the Wbl-4 element (Figure 9A, W4D). These results indicate that recombinant *BjPHL2a* proteins could interact with the Wbl-4 element.

### 2.10. BjPHL2a Activates a Mini-Promoter Containing a Wbl-4 Element In Vivo

**Figure 10:**
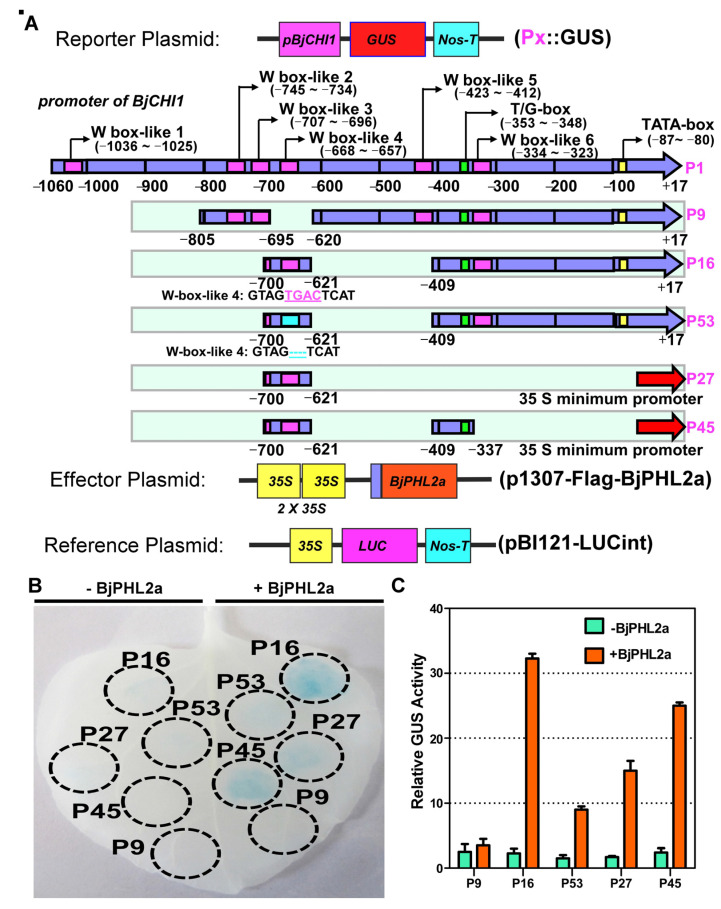
*BjPHL2a* binds to the Wbl-4 element *in vivo*. (**A**) Schematic diagram of the reporter and the effector constructs used in co-transfection experiments. For the reporter construct, the deletion- and mutation-derivatives of the *BjCHI1* promoter (P9, P16, P27, P45, and P53) were fused with GUS. For the effector construct, 2 × 35S is a tandem repeat of the cauliflower mosaic virus (CaMV) 35S promoter that is used to express *BjPHL2a* in tobacco leaves. (**B**) Transactivation activity of the reporter constructs based on *BjPHL2a* expression. Tobacco leaves co-infiltrated with the indicated constructs were stained with X-Gluc for *GUS* expression analysis. The infiltration areas are indicated with dashed cycles. (**C**) Quantization of GUS activity for every construct in transiently transformed tobacco leaves. The GUS activity was normalized to LUC activity. Columns show the ratio of GUS activity induced by *BjPHL2a* to that of no induction. Values represent means ± SD from three replicates.

To further analyze whether *BjPHL2a* actually had transactivation activity in vivo, we sought to directly investigate its role in transactivating the Wbl-4 element by transient expression in *Nicotiana benthamiana*. As previously described, we also recruited several key deletion derivatives of the *BjCHI1* promoter, such as P9, P16, P53, P27, and P45 (Figure 9A) as promoters for *GUS* fusion reporter plasmids [14,15]. Consistent with previous reports [14,15], *BjPHL2a* activated the expression of *GUS* driven by P16-, P27-, and P45 promoters containing Wbl-4 box, but not by P53 and P9 (Figure 10B,C). Compared with P16, only the TGAC in the core sequence of the Wbl-4 element was absent in P53 and the Wbl-4 box was removed in P9. Therefore, these observations indicate that *BjPHL2a* can also transactivate the Wbl-4 element in vivo.

## 3. Discussion

The MYB-like superfamily transcription factors are one of the largest transcription factor families in plants and play important roles in a wide range of physiological processes, such as light signaling, the anthocyanin biosynthetic pathway, hypocotyl elongation, phosphate starvation, and resistance to biotic and abiotic stresses [29,34]. Although *MYB* and *MYB-CC* transcription factor genes have been identified in a number of plant species at the genome-wide level [16,17,23,24,25,26,27,28,29,30,31,32,33,34,35], there is little information available on this superfamily in the *Brassica juncea* genome. Recently, bioinformatics and publicly released data sets describing the genome and transcriptome of *Brassica juncea* have paved the way for systemically exploring this important crop species [1,37,38]. In the present study, a total of 502 *BjMYB* genes were identified in *Brassica juncea*, which were divided into six subfamilies (1R-, R2R3-, 3R-, 4R-, atypical MYBs, and MYB-CCs) and clustered into 15 groups (Figure 1 and Table S1). The number of *BjMYB* genes was greater than those of *Arabidopsis thaliana* (212) [24,39], *Oryza sativa* (213) [24,40], *Zea mays* (169) [17,23], *Brassica rapa* (354), and *Brassica oleracea* (330), but fewer than that in *Brassica napus* (556) [24,26,27]. BjMYB proteins were not equally distributed into 15 different groups in the phylogenetic tree (Figure 1). Among them, the top largest groups (C1, C9, C13, C14, and C15) represented around 70% of the total BjMYB members (Figure 1 and Table S1), suggesting that these groups may be highly differentiated in the *Brassica juncea* genome. Moreover, the large range of Mw and pI of BjMYBs suggests their functional diversity in distinct biological processes, which may be attributed to their difference in full-length amino acid sequences and rest domains, in addition to similarity in MYB domains.

As displayed in Figure 2, the 471 (93.82%) identified members of *BjMYB* genes were unevenly distributed across all 18 chromosomes at different densities. Among them, 241 *BjMYB* genes were mapped to the A subgenome and 230 *BjMYB* genes to the B subgenome of *Brassica juncea*. The chromosomes A01 to A10 and B01 to B08 represent the two parental diploid ancestors of *Brassica juncea*. The number of *BjMYB* genes (502) was found to be less than the sum of its two parental lines, *Brassica rapa* (301) and *Brassica nigra* (>300) [26,27], indicating loss of *MYB* genes also happened after allopolyplodization. In general, every AtMYB-CC was expected to have four to six homologs in *Brassica juncea*. Consistently, 15 *AtMYB-CCs* had 64 homologous genes in *Brassica juncea* (Figure 4A and Table S1). For example, *AtPHL2* had four homologs (*BjPHL2a, BjPHL2b, BjPHL2a1*, and *BjPHL2b1*) and *AtPHR1* had five homologs (*BjPHR1a, BjPHR1b, BjPHR1a1, BjPHR1b1*, and *BjPHR1b2*). In terms of the overall gene structure, significant differences were found in the number of introns among *BjMYB-CC* subfamily members (Figure 3). However, the overall intron/exon distribution within members of the same subclade was highly similar, such as PHL2-, PHL3-, and PHR1-subclade (Figure 3), indicating that the biological significance of these homologous genes may be redundantly conserved to a certain extent.

It is tempting to speculate that gene expression patterns can provide important clues for gene function [33,36]. The expression patterns of 64 *BjMYB-CC* genes from three type of abiotic stresses were determined (Figure 5). Only a few BjMYB-CC genes displayed inducible expression by at least one analyzed treatment (Figure 5 and Table S4). Furthermore, qRT-PCR analysis revealed similar expression patterns after *Botrytis cinerea* infection of the three *AtPHL2* homologs in *Brassica juncea* (*BjPHL2a, BjPHL2b,* and *BjPHL2a1*, Figure 6).

Previous work has shown that the MYB-CC transcription factor BjMYB1 can bind to W-box or W-box-like elements, but not to AC elements [15]. As mentioned above, MYB–CC type transcription factors are a distinct subfamily of the plant MYB superfamily, which were originally identified as phosphate starvation response (PHR) protein family transcription factors, such as AtPHR1 and OsPHR2 [20]. It is well known that PHR protein family members can transactivate the expression of a broad range of PSI genes by binding to P1BS *cis*-elements in a phosphate (Pi)-dependent manner [17,20]. The P1BS *cis*-regulatory element is characterized by the nucleotide motif GNATATNC or CATATATG [20,41]. We also observed a putative P1BS-like *cis*-element (CATATATCG) near the fungus-responsive Wbl-4 box in the promoter of *BjCHI1*. Besides serving as master transcriptional regulators of the phosphate sensing pathway [22,39,42], PHR1-like MYB-CC proteins (such as AtPHR1 and OsPHR1/2/3) have been recently reported to play central roles in the balance between nutrient stress response and immune regulation or mycorrhizal symbiosis with respect to root microbiota [19,43,44]. One possibility is that the observed binding activity of MYB-CC-like proteins (BjMYB1 and *BjPHL2a*) to the Wbl-4 box containing mini-promoter may also be partially attributed to the putative P1BS-like *cis*-element near the Wbl-4 box. In other words, PHR1-like MYB-CC proteins may also have roles in fighting against *Botrytis cinerea* infection via modulating the expression of *BjCHI1*. We found four AtPHL2 homologs in *Brassica juncea* and similar expression patterns after *Botrytis cinerea* infection was observed among three paralogs (Figure 6). The previously isolated BjMYB1 showed sequence differences compared with other BjPHL2 s (Figures S1–S3). Therefore, it is possible that *BjPHL2a*, together with the closed paralogs, may be redundantly involved in fighting against fungal attack by interacting with the Wbl-4 element in the promoter of *BjCHI1*.

Recently, hierarchical and dynamic regulation of defense-responsive genes by AtMYB51 and AtWRKY33 has been reported [45]. The cognate nucleotide motif of the Wbl-4 box (GTGACT), also shows a minor difference to the core nucleotide sequence of classical W-boxes (C/TTGACC/T) [15,46]. This may be the major reason that we have not isolated any WRKY-like transcription factors to date. However, another possibility is that *BjPHL2a* or BjMYB1 and BjWRKY transcription factors may function coordinately to control the inducible expression of *BjCHI1*.

## 4. Materials and Methods

### 4.1. Identification of the MYB Gene Superfamily in Brassica juncea

Protein sequences of *Brassica juncea* var. *tumida* inbred line (T84−66, GenBank accession number: LFQT00000000, BioProject PRJNA285130) were obtained from the BGD (http://brassicadb.bio2db.com/) [38,47]. HUMMER3.1 software (Linux version) was used to isolate *BjMYBs* from *Brassica juncea* var. *tumida* inbred line T84−66 through the HMM profile corresponding to Myb-like DNA binding domain (PF00249, PF08914, PF13921, PF12776, PF13873, PF15963, PF13837) and MYB-CC domain (PF14379), with the threshold set at *E*-value < 1 × 10^–10^. A total of 195 AtMYB [24] and 15 MYB-CC protein sequences were downloaded from TAIR (www.arabidopsis.org) and were used as queries to perform a BLASTP search in the local protein database of *Brassica juncea* var. *tumida* inbred line (T84−66). Then, the putative BjMYBs were obtained via taking the intersection of the HUMMER and BLASTP methods. Finally, these proteins were submitted to the NCBI-CDD server (http://www.ncbi.nlm.nih.gov/Structure/cdd/wrpsb.cgi) and the SMART (Simple Modular Architecture Research Tool, http://smart.embl-heidelberg.de/) database to perform the Myb-like DNA binding domain or MYB-CC domain predictions as previously described [48]. The theoretical molecular weight and isoelectric points of BjMYBs were calculated by DNAstar as previously described [49].

### 4.2. Phylogenetic, Chromosome Distribution, Gene, and Protein Structure Analysis

Protein sequences of the BjMYB members were first aligned with the MUSCLE tool. The phylogenetic tree was then generated using MEGA 11 with the neighbor-joining (NJ) method (bootstrap replications, n = 1000) as previously described [36,49]. The phylogenetic tree was then visualized using Figtree software (version 1.4.4) [36,48]. The physical positional information of each *BjMYB* gene was downloaded from NCBI (accession number PRJNA285130) [38]. Locations of *BjMYB* genes on *Brassica juncea* chromosomes were then deciphered with the MapChart tool as previously described [36]. The overall intron/exon organization of the *MYB-CC* genes was determined based on GFF annotation files with Gene Structure View (Advanced) from TB tools as previously described [48]. Protein sequences of 15 AtMYB-CCs and 64 BjMYB-CCs were aligned using the MUSCLE tool, and the maximum likelihood trees were generated using MEGA 11, as mentioned above. The conserved motifs and protein architecture were predicted by the MEME (Multiple Em for Motif Elicitation) tool (http://meme-suite.org/tools/meme) as previously described [36].

### 4.3. Expression Profiles of the BjMYB-CC Genes

RNA-seq data from *Brassica juncea* under drought, high temperature, and salt stresses were downloaded from GEO (Gene Expression Omnibus, accession numbers GSE66389 and GSE64242) [37]. All RNA-seq data were mapped to the reference genome of *Brassica juncea* with HISAT2 software. Transcript abundance of the *BjMYB-CC* genes were calculated using TPM (transcripts per million) values generated with the FeatureCounts R package and a histogram was generated using TBtools software as previously described [48].

### 4.4. Plant Materials and Growth Conditions

Plants of *Nicotiana benthamiana* and *Brassica juncea* were grown in a growth chamber at 25 °C (light)/22 °C (dark) under a 16 h light/8 h dark cycle. *Nicotiana benthamiana* was used to carry out transient expression assays and *Brassica juncea* was used for constructing a cDNA library and endogenous gene expression assays.

### 4.5. Plasmid Construction

To express *BjPHL2a* in yeast, the full-length CDS of *BjPHL2a* was cloned into the pGADT7 vector between the *Bam*HI and *Sal*I sites. The following primer pairs were used for PCR amplification: *BjPHL2a*-2bp*Bam*F: 5′-CGGGATCCGTATGTACTCGGCGATTCGCTC-3′ and *BjPHL2a*-1bp*SalR*: 5′-ACGCGTCGACATCCCATGGTACTACCCGGCACAG-3′ (*Bam*HI and *Sal*I sites are underlined).

To produce FLAG-tagged *BjPHL2a*, the CDS of *BjPHL2a* was amplified with the following primers: *BjPHL2a*-*Bam*F: 5′-CGGGATCCATGTACTCGGCGATTCGCTC-3′ and *BjPHL2a*-*SalR*: 5′-ACGCGTCGACTCCCATGGTACTACCCGGCACAG-3′ (*Bam*HI and *Sal*I sites are underlined). The sequence-confirmed full-length CDS of *BjPHL2a* was then cloned into the *Bam*HI and *Sal*I sites of the binary expression vector p1307-3Flag.

To make YFP tagged *BjPHL2a* (YFP-*BjPHL2a*), the CDS was removed from p1307-3Flag-*BjPHL2a* and inserted into the pCM1205-YFP vector between the *Bam*HI and *Sal*I sites, resulting in an N-terminal fusion to YFP.

To obtain His-tagged *BjPHL2a* recombinant protein, the CDS was removed from p1307-Flag-*BjPHL2a* and inserted into pET-28a (Novagen) between the *Bam*HI and *Sal*I sites.

### 4.6. Yeast One-Hybrid Assay

Yeast one-hybrid assays were performed as previously described [15]. Briefly, pGADT7-*BjPHL2a* plasmids or pGADT7 empty vector were transformed into the bait-reporter yeast strains Y1Hgold [pBait-AbAi] or Y1Hgold [pBait-m-AbAi] by the lithium acetate/single-stranded carrier DNA/polyethylene glycol method [50,51]. Transformed yeast cells were selected on SD/-Leu (no AbA) medium. DNA binding and transactivation were determined by checking the growth of transformed yeast cells with serial dilutions on SD/-Leu/+AbA^550^ (SD/-Leu media containing 550 ng/mL AbA) agar plates for 2~3 days. Growth was recorded by photography.

### 4.7. Subcellular Localization of BjPHL2a

The YFP-*BjPHL2a* fusion vector (pCM1205-YFP-*BjPHL2a*) was transiently expressed in *Nicotiana benthamiana* leaves by agroinfiltration as previously described [15,50,51]. Briefly, the *Agrobacterium tumefasciens* EHA105 harboring pCM1205-YFP-*BjPHL2a* was grown on agar-LB containing 100 µg/mL chloramphenicol and 30 µg/mL rifampicin, then suspended in MMA buffer (10 mM MgCl_2_, 10 mM MES pH 5.5, 100 µM acetosyringone) at OD_600_ 0.5 and incubated at 28 °C for 3 h. The fifth and sixth expanded leaves of *Nicotiana benthamiana* were infiltrated at the blade back with a 1 mL needleless syringe. YFP fluorescence of cells from the lower epidermis was observed with a confocal laser scanning microscope 2~3 days after infiltration [15].

### 4.8. Protein Purification and Electrophoretic Mobility Shift Assay (EMSA)

Recombinant protein purification and an EMSA assay were performed as previously described [15]. Briefly, the expression of recombinant His-tagged *BjPHL2a* fusion protein was induced with 0.6 mM isopropyl-D-thiogalactoside (IPTG) until the OD_600_ reached 0.5. Recombinant His-BjPHL2a protein was purified with Ni^2+^ affinity resin (Ni-NTA, QIAGEN) according to the manufacturer’s instructions. Purified His-BjPHL2a was used for the EMSA assay as previously described [15]. Complementary pairs of non-labeled and 3′-biotin-labeled oligonucleotides of W4 (intact Wbl-4 element), W4D (core sequence TGAC in the Wbl-4 element deleted), and W5 (W-box-like 5 elements) were synthesized and used as probes. The following probe sequences were used: W4: 5′-gcccagctagtgagtagtgactcatgaggtagagagaggg-3′, W4D: 5′-gcccagctagtgagtagtcatgaggtagagagaggg-3′, and W5: 5′-agggtggtccctctagtgactcatgagctagagagagggt-3′ (the core W box-like elements are underlined).

### 4.9. Histochemical Staining of GUS and GUS Quantitative Assays

GUS staining and GUS quantitative analysis was performed as described in a previous study [14]. Briefly, the agrobacterium strain EHA105 containing the GUS constructor and its derivatives (P16 and P53) were co-infiltrated with *BjPHL2a*, and with pBI121-LUCint in *Nicotiana benthamiana* leaves, and transiently expressed. For histochemical staining of GUS, the infiltrated *Nicotiana benthamiana* leaves were stained and photographed. For the GUS quantitative assay, the infiltrated *Nicotiana benthamiana* leaf discs were cut and used for GUS and LUC assay as described previously [14].

### 4.10. Determination of the Expression of BjPHL2a and its Paralogs after Botrytis cinerea Infection

*Botrytis cinerea* was cultured on potato agar media plus 1.5% dextrose (potato 200 g /L, glucose 20 g/L, agar 15 g/L, pH 6.0) at 22 °C for 14 days. The conidia of well-grown *Botrytis cinerea* were suspended in sterile distilled water, filtered with two layers of gauze, and diluted to 5 × 10^5^ cells per milliliter. To determine the expression level of *BjPHL2a* and its paralogs after *Botrytis cinerea* infection, 16-day-old *Brassica juncea* plants were inoculated with the conidial suspension by spraying and sampled at 0, 18, 24, 36, 48, 72, and 96 hours after infection. qRT-PCR was performed using SYBR Premix Ex TaqTM II (TAKARA BIO Inc., Shiga, Japan) on a 7500 Fast Real-Time PCR System (Applied Biosystems, Foster City, CA, USA) with the following conditions: 95 °C for 1 min, followed by 40 cycles of 95 °C for 10 s and 60 °C for 34 s in 20 μL reaction volumes. A melting curve was generated for each reaction to ensure specific amplification. The relative expression was quantified using the comparative 2^−ΔΔCT^ method. Primer pairs BjPHL2aF1/ BjPHL2aR1 (BjPHL2aF1: 5′-GGAACAGAACGAGAGTTACC-3′ and BjPHL2aR1: 5′-CTTCCCTTGTGCTTCTATCC-3′), BjPHL2a1F1/BjPHL2a1R1 (BjPHL2a1F1: 5′-CAGAACGAGGGTTACCAAGTC-3′ and BjPHL2a1R1: 5′-TCCTTGTGCCTCTATCCTCAG-3′), BjPHL2bF1/BjPHL2bR1 (BjPHL2bF1: 5′-AGCAACATCACGACCAACTG-3′ and BjPHL2bR1: 5′-TCGCTTCATACCCAACACTG-3′), and BjPHL2b1F1/BjPHL2b1R1 (BjPHL2b1F1: 5′-AGCAGTCCCGTTCTTAGATG-3′ and BjPHL2b1R1: 5′-CCGAACAGTTGGTTGTGATG-3′) were used for qRT-PCR analysis of *BjPHL2a*, *BjPHL2a1*, *BjPHL2b*, and *BjPHL2b1,* respectively. Primer pairs BjActin-F/ BjActin-R of Actin gene in *Brassica juncea* (BjuB025449, an AtACT2-like gene in *Brassica juncea*) were used as internal references (BjActin-F: 5′-CTTCTTACCGAGGCTCCTCT-3′ and BjActin-R: 5′-AAGGATCTTCATGAGGTAATCAGT-3′) as previously described [15].

## 5. Conclusions

In the present study, the identification, classification, and expression analysis of the *MYB* gene superfamily was comprehensively evaluated at the whole genome level in *Brassica juncea*. Functional and molecular studies showed that *BjPHL2a* functions as a transcription activator by interacting with the Wbl-4 element in the promoter of *BjCHI1* for targeted gene inducible expression after *Botrytis cinerea* infection.

## Data Availability

Not applicable.

## Supplementary Materials

The following are available online at https://www.mdpi.com/article/10.3390/plants12051011/s1. Table S1. Details of the identified *BjMYB* genes in *Brassica juncea*; Table S2. Sequence information of additional conserved motifs identified from BjMYB proteins in *Brassica juncea*; Table S3. Sequence information of the conserved MYB-CC domain from AtMYB-CC and BjMYB-CC proteins; Table S4. Expression of *BjMYB-CCs* under different stresses; Figure S1. Multiple sequence alignment of BjPHL2 subclade members; Figure S2. Sequence alignment of BjMYB1 and BjPHL2b1 at the CDS level; Figure S3. Sequence alignment of BjMYB1 and BjPHL2b1 at the protein level; Figure S4. Protein architecture of BjMYBs.
