# Supplementary material for: Genome-Wide Analysis of MYB Transcription Factor Gene Superfamily Reveals BjPHL2a Involved in Modulating the Expression of BjCHI1 in Brassica juncea
Source: Plants (Basel). 2023 Feb 23;12(5):1011. doi: 10.3390/plants12051011 (PMC10004776; doi:10.3390/plants12051011)
Supplement: Supplementary file 1 [file plants-12-01011-s001.zip › Figures S1-S4.pdf]

Figure S1.

```
BjuA005498-BjPHL2a1     MY-SAIRSLPLDGGDYHGPLDGTNLPGDACLVLTTDPKPRLRWTAELHERFVDAVTQLGG 59
BjuB039462-BjPHL2b1     MYSAAIRSLPLD-GDYHGPLDGTNLPGDACLVLTTDPKPRLRWTAELHERFVDAVTQLGG 59
BjMYB1                  ------------------------------------------------------------ 0
BjuA012774-BjPHL2a      -MYSAIRSLPLDGGEYHGPLDGTNLPGDACLVLTTDPKPRLRWTAELHERFVEAVTELGG 59
BjuB032221-BjPHL2b      -MYSAIRSLALDGGEYHGPLDGTNLPGDACLVLTTDPKPRLRWTAELHEKFVEAVTELGG 59


BjuA005498-BjPHL2a1     PDKATPKTIMRTMGVKGLTLYHLKSHLQKFRLGRQACKDSTDNS-KDASCVGESQDTGSS 118
BjuB039462-BjPHL2b1     PEKATPKTIMRTMGVKGLTLYHLKSHLQKFRLGRQACKESTENS-KDASCVGESQDTGSS 118
BjMYB1                  ------------MGVKGLTLYHLKSHLQKFRLGRQACKESTENS-KDASCVGESQDTGSS 47
BjuA012774-BjPHL2a      PEKATPKTLMRTMGVKGLTLYHLKSHLQKFRQGRQACKESTDNSNKDASCVGESQDTGSS 119
BjuB032221-BjPHL2b      PEKATPKTLMRTMGVKGLTLYHLKSHLQKFRQGRQACKESTDNS-KDASCVGESQDTGSS 118
                                    ******************* ******:**:** ***************

BjuA005498-BjPHL2a1     SSSSLRMAAQEQNEGYQVTEALRAQMEVQRRLHEQLEHGQVQRRLQLRIEAQGKYLQSIL 178
BjuB039462-BjPHL2b1     SSSSLRMAAQEQNEGYQVTEALRAQMEVQRRLHEQLE---VQRRLQLRIEAQGKYLQSIL 175
BjMYB1                  SSSSLRMAAQEQNEGYQVTEALRAQMEVQRRLHEQLEYGQVQRRLQLRIEAQGKYLQSIL 107
BjuA012774-BjPHL2a      SPSSLKLAAQEQNESYQVTEALRAQMEVQRRLHEQLE---VQRRLQVRIEAQGKYLQSIL 176
BjuB032221-BjPHL2b      SPSSLRLAAQEQNEGYQVTEALRAQMEVQRRLHEQLE---VQRRLQVRIEAQGKYLQSIL 175
                        * ***::*******.**********************   ******:*************

BjuA005498-BjPHL2a1     EKACKAFDDQAAAFVGLEAAREELSELAIKVSNSSQGT-AVPFFDTTKM-MMMPSLSELA 236
BjuB039462-BjPHL2b1     EKACQAFDDQAAAFVGLEAAREELSELAIKVS---QGT-AVPFLDATKM-MMMPSLSELE 230
BjMYB1                  EKACQAFDDQAAAFVGLEAAREELSELAIKVS---QGT-AVPFLDATKM-MMMPSLSELE 162
BjuA012774-BjPHL2a      EKACKAFDEQAAMFTGLETAREELSELAIKVSNSSQGA-TVPYFDATKMMMMMPSLSELE 235
BjuB032221-BjPHL2b      EKACKAFEEQAVTFGGLETAREELSDLAIKASSSSQGTTTVPYFDATKMMMMMPSLSELE 235
                        ****:**::**. * ***:******:****.*   **: :**::*:*** *********

BjuA005498-BjPHL2a1     VAV-DTK-NNITTNCSVESSLTSNTNGSSVSAASMKKRLRGDDVGLGYEAGWNVPSSSTI 294
BjuB039462-BjPHL2b1     VAI-DTK-NNITTNCSVESSLTSNTNGSSVSAASMKKRHRGDDVGLGYEAGWIVPSSTIG 288
BjMYB1                  VAI-DTK-NNITTNCSVESSLTSNTNGSSVSAASMKKRHRGDDVGLGYEAGWIVPSSTIG 220
BjuA012774-BjPHL2a      VAAIDHKSNITTTNCSVESSLTSNTNGSSVSAASMKKRHRGGD-NVGYEGSWTVPGSTMG 294
BjuB032221-BjPHL2b      VAAIDQKSNIT-TNCSVESSLTSNTNGSSVSAASMKKRHRG-D-SVGYEASWIMPSSTIG 292
                        **  * * *   ************************** ** * .:***..* :*.*:

BjuA005498-BjPHL2a1     G              295
BjuB039462-BjPHL2b1     -              288
BjMYB1                  -              220
BjuA012774-BjPHL2a      -              294
BjuB032221-BjPHL2b      -              292
```

Figure S1. Multiple sequence alignment of BjPHL2 subclade members.

The protein sequences of all BjPHL2 subclade members are aligned with

Clustal Omega (https://www.ebi.ac.uk/Tools/msa/clustalo/). The 71-amino acid-long deletion at the N-terminal and a YGQ insertion in the central between BjMYB1 and BjPHL2b1 are indicated in yellow box.

Figure S2.

```
BjMYB1               ------------------------------------------------------------ 0
BjuB039462-BjPHL2b1  ATGTACTCGGCGGCGATTCGGTCGCTTCCTCTCGACGGTGACTACCATGGACCTCTTGAC 60


BjMYB1               ------------------------------------------------------------ 0
BjuB039462-BjPHL2b1  GGTACTAATCTTCCCGGCGACGCCTGTTTGGTCTTAACCACTGACCCCAAACCCCGTCTT 120


BjMYB1               ------------------------------------------------------------ 0
BjuB039462-BjPHL2b1  CGGTGGACCGCGGAGCTCCACGAGAGGTTCGTTGACGCCGTCACGCAGCTCGGTGGTCCC 180


BjMYB1               ---------------------------------ATGGGAGTGAAAGGCCTCACCCTCTAC 27
BjuB039462-BjPHL2b1  GAAAAAGCGACGCCCAAAACTATCATGAGAACAATGGGAGTGAAAGGCCTCACCCTCTAC 240
                                                      ***************************

BjMYB1               CACCTCAAATCACATCTCCAGAAATTCCGGCTAGGGAGGCAAGCTTGTAAAGAATCAACT 87
BjuB039462-BjPHL2b1  CACCTCAAATCACATCTCCAGAAATTCCGGCTAGGGAGGCAAGCTTGTAAAGAATCAACT 300
                     ************************************************************

BjMYB1               GAGAACTCCAAGGATGCTTCTTGTGTTGGGGAGAGTCAGGACACAGGTTCATCTTCATCG 147
BjuB039462-BjPHL2b1  GAGAACTCCAAGGATGCTTCTTGTGTTGGGGAGAGTCAGGACACAGGTTCATCTTCATCG 360
                     ************************************************************

BjMYB1               TCATCACTGAGAATGGCAGCGCAGGAGCAGAACGAGGGTTACCAAGTCACTGAAGCTCTA 207
BjuB039462-BjPHL2b1  TCATCACTGAGAATGGCAGCGCAGGAGCAGAACGAGGGTTACCAAGTCACTGAAGCTCTA 420
                     ************************************************************

BjMYB1               CGCGCTCAAATGGAAGTCCAAAGAAGACTACACGAGCAATTGGAGTATGGGCAGGTACAA 267
BjuB039462-BjPHL2b1  CGCGCTCAAATGGAAGTCCAAAGAAGACTACACGAGCAATTGG---------AGGTACAA 471
                     *******************************************         ********

BjMYB1               CGGAGACTCCAGCTGAGGATAGAGGCACAAGGAAAGTACTTACAATCGATCCTTGAGAAA 327
BjuB039462-BjPHL2b1  CGGAGACTCCAGCTGAGGATAGAGGCACAAGGAAAGTACTTACAATCGATCCTTGAGAAA 531
                     ************************************************************

BjMYB1               GCTTGCCAGGCCTTTGACGACCAAGCTGCTGCTTTTGTTGGGCTCGAGGCAGCTAGGGAA 387
BjuB039462-BjPHL2b1  GCTTGCCAGGCCTTTGACGACCAAGCTGCTGCTTTTGTTGGGCTCGAGGCAGCTAGGGAA 591
                     ************************************************************

BjMYB1               GAGCTATCAGAGCTAGCCATCAAAGTGTCTCAAGGAACAGCAGTCCCGTTCTTAGATGCA 447
BjuB039462-BjPHL2b1  GAGCTATCAGAGCTAGCCATCAAAGTGTCTCAAGGAACAGCAGTCCCGTTCTTAGATGCA 651
                     ************************************************************
```

```
BjMYB1                  ACAAAGATGATGATGATGCCATCTTTGTCTGAGCTTGAAGTAGCGATAGACACCAAAAAC 507
BjuB039462-BjPHL2b1     ACAAAGATGATGATGATGCCATCTTTGTCTGAGCTTGAAGTAGCAATAGACACCAAAAAC 711
                        ******************************************** ***************

BjMYB1                  AACATCACAACCAACTGTTCGGTTGAAAGCTCTCTGACTTCCAACACCAATGGGAGCTCG 567
BjuB039462-BjPHL2b1     AACATCACAACCAACTGTTCGGTTGAAAGCTCTCTGACTTCCAACACCAATGGGAGCTCG 771
                        ************************************************************

BjMYB1                  GTTTCTGCTGCATCGATGAAGAAGCGGCATCGTGGAGACGATGTAGGCCTAGGGTACGAG 627
BjuB039462-BjPHL2b1     GTTTCTGCTGCATCGATGAAGAAGCGGCATCGTGGAGACGATGTAGGCCTAGGGTACGAG 831
                        ************************************************************

BjMYB1                  GCAGGGTGGATTGTGCCTAGTAGTACCATTGGATAA                         663
BjuB039462-BjPHL2b1     GCAGGGTGGATTGTGCCTAGTAGTACCATTGGA---                         864
                        *********************************
```

Figure S2. Sequence alignment of BjMYB1 and BjPHL2b1 at the CDS level. The CDS sequences of BjMYB1 and BjPHL2b1 are aligned with Clustal Omega. The observed 213 nt absence in the 5' end, 9 nt insertion in the central and a G to A replacement at 3’ end between BjMYB1 and BjPHL2b1 are indicated by yellow box.

Figure S3.

```
BjuB039462-BjPHL2b1   MYSAAIRSLPLDGDYHGPLDGTNLPGDACLVLTTDPKPRLRWTAELHERFVDAVTQLGGP 60
BjMYB1                ------------------------------------------------------------ 0


BjuB039462-BjPHL2b1   EKATPKTIMRTMGVKGLTLYHLKSHLQKFRLGRQACKESTENSKDASCVGESQDTGSSSS 120
BjMYB1                -----------MGVKGLTLYHLKSHLQKFRLGRQACKESTENSKDASCVGESQDTGSSSS 49
                                 *************************************************

BjuB039462-BjPHL2b1   SSLRMAAQEQNEGYQVTEALRAQMEVQRRLHEQLE---VQRRLQLRIEAQGKYLQSILEK 177
BjMYB1                SSLRMAAQEQNEGYQVTEALRAQMEVQRRLHEQLEYGQVQRRLQLRIEAQGKYLQSILEK 109
                      ***********************************   **********************

BjuB039462-BjPHL2b1   ACQAFDDQAAAFVGLEAAREELSELAIKVSQGTAVPFLDATKMMMMPSLSELEVAIDTKN 237
BjMYB1                ACQAFDDQAAAFVGLEAAREELSELAIKVSQGTAVPFLDATKMMMMPSLSELEVAIDTKN 169
                      ************************************************************

BjuB039462-BjPHL2b1   NITTNCSVESSLTSNTNGSSVSAASMKKRHRGDDVGLGYEAGWIVPSSTIG          288
BjMYB1                NITTNCSVESSLTSNTNGSSVSAASMKKRHRGDDVGLGYEAGWIVPSSTIG          220
                      ***************************************************
```

Figure S3. **Sequence alignment of BjMYB1 and BjPHL2b1 at the protein level.** The protein sequences of BjMYB1 and BjPHL2b1 are aligned with Clustal Omega. The observed 71-amino acid-long deletion at the N-terminal and a YGQ insertion in the central between BjMYB1 and BjPHL2b1 are indicated in yellow box.

Figure S4.

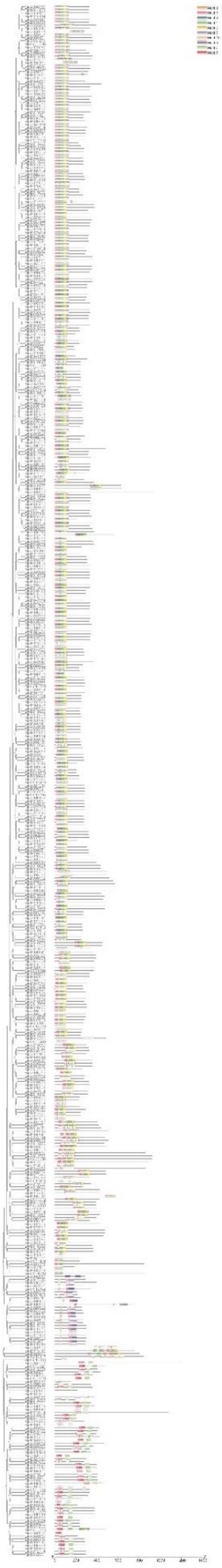

Figure S4. Protein architecture of BjMYBs. The distribution of conserved motifs identified from 502 BjMYB proteins are predicted using the MEME (Multiple Em for Motif Elicitation) tool. Each motif is represented by a number in colored box. See Table S2 for detailed motif information.
